# Development of a Neutral Mine Drainage Prediction Method Using Modified Kinetics Tests and Assessment of Sorption Capacities

**DOI:** 10.1007/s10230-025-01023-6

**Published:** 2025-01-21

**Authors:** Vincent Marmier, Benoît Plante, Isabelle Demers, Mostafa Benzaazoua

**Affiliations:** 1 Research Institute on Mine and Environment, Université du Québec en Abitibi-Témiscamingue (UQAT), Rouyn-Noranda, Québec Canada; 2 Geology & Sustainable Mining Institute (GSMI), University Mohamed IV Polytechnique (UM6P), Ben Guerir, Morocco

**Keywords:** Modified kinetic column tests, Sorption tests, Risk assessment, Metal leaching

## Abstract

Prediction of neutral mine drainage (NMD) chemistry is difficult with the predictive tools developed for acid mine drainage (AMD). To address this problem, a methodology to assess NMD risk was developed using Lac Tio Mine waste rock as a positive control. The methodology compares the maximum potential for contaminant release (in this case, nickel) using the waste rock’s total metal content and the sorption capacity of the material (q_max_) combined with a mineralogical assessment and modified kinetic leaching experiments that use a chelating agent to prevent immobilization processes from occurring. The results indicate that the potential NMD risk associated with Lac Tio waste rock would be assessed as probable with the proposed methodology. Indeed, the total nickel concentrations in the Lac Tio waste rock range from 270 to 590 mg/kg. The nickel is found in Ni-rich pyrites, which proved to effectively leach when no immobilization occurred (using ethylenediaminetetraacetic acid, or EDTA, leaching). The material’s sorption capacities were between 127 and 197 mg/kg of Ni. The sorption capacity to total Ni content ratio of the material was < 1, indicating that the material has fewer sorption sites for Ni than Ni contained within the material, thereby underscoring the potential risk of Ni leaching over time. The approach proposed in this work provides an additional tool for the assessment water quality risk associated with NMD.

**Supplementary Information:**

The online version contains supplementary material available at 10.1007/s10230-025-01023-6.

## Introduction

Mining activities have been increasing in response to the growing demand for metal/mineral resources. As a result, mining claims have more than doubled in Québec since the 2000s, and total investments in exploration and mining infrastructures have tripled (Institut de la statistique du Québec 2016). The increased mining activity can adversely affect the environment if responsible waste management approaches are neglected. In the past, mine wastes were often left on sites after exploitation without consideration of their physical and geochemical behaviors or the environmental effects they might have. Such mining activities have resulted in many contaminated sites that are now under government responsibility for their surveillance and eventual remediation (Gouvernement du Québec 2023). To prevent sites from becoming a public financial liability as a result of bankruptcy, the Québec government requires mining companies to provide an extensive assessment of the mining project’s financial feasibility, remediation and waste management plans, an environmental impact assessment, and financial guarantees to cover the cost of reclamation of their mine sites.

Mining generates wastes with low economic value due to residual metals lost or not valorized. These mine wastes come in two main forms: waste rock from ore excavation and tailings from ore processing of primarily sulfide orebodies (Bussière and Guittonny 2020).

For long-term geochemical risk assessment of mine wastes, it is crucial to consider the potential for acid generation (PAG), through the well-known acid-base accounting (ABA) methods. Iron-containing sulfides, which are frequently found in many mining wastes, produce acidity when exposed to the atmosphere (Blowes et al. 2003; Kirby and Cravotta III 2005; Nordstrom et al. 2015). To assess the risk of acid generation, static and kinetic methods were developed (e.g. Bouzahzah et al. 2014; MEND 2009; Plante et al. 2020). These methods compare the acid generation capacity (or potential) to the neutralizing capacity (or potential) of other minerals, such as carbonates and silicates. The ABA assessment is crucial in determining whether the mine wastes are likely to form acid mine drainage (AMD), which is a mine effluent characterized by a pH below 6 and high concentrations of sulfate, metals, and other dissolved species that have harmful effects on the environment (e.g. Dubé et al. 2005; Rozon-Ramilo et al. 2011; Steyn et al. 2019).

However, in some cases, mine wastes may not generate acidity due to low sulfide concentrations and/or high neutralization potential, resulting in an effluent or leachate with a near-neutral pH (e.g. Heikkinen and Räisänen 2009; Heikkinen et al. 2009; Nicholson and Rinker 2000). Despite the neutral pH, there is still a risk of contaminant concentrations exceeding regulatory limits and/or adversely affecting the receiving environment (MDDEP 2012). These mine drainage waters are commonly referred to as neutral mine drainage (NMD). Prediction of mine drainage chemistry is done systematically during the project evaluation phase, but NMD is difficult to predict, and new predictive tools are needed.

Predicting NMD is challenging because of the lag time required to release metals and other contaminants in mined materials that are not potentially acid generating (Plante et al. 2011a). Indeed, the metals released at near-neutral pH can be controlled temporarily by sorption phenomena (Heikkinen et al. 2009; Plante et al. 2010). To date, kinetic AMD prediction methods, such as weathering cells (e.g. Villeneuve et al. 2003), humidity cells (ASTM 2013), and leaching columns (Lawrence et al. 1989) have been used to predict NMD, but these methods have not yet yielded conclusive results for NMD prediction. When sorption controls the water quality by preventing metals from leaching out during the kinetic tests, metal leaching will only be detected after saturation of sorption sites, which can take more time than the duration of most of the traditionally used kinetic tests (from 10 to 20 weeks to a few years; Bouzahzah et al. 2014; MEND 2009; Plante et al. 2020). Therefore, NMD prediction can be challenging using these methods when sorption controls water quality, as is the case for the Lac Tio waste rock (Demers et al. 2013; Pepin 2009; Plante et al. 2010, 2011a, b, 2014, 2015; Poaty et al. 2021), and better predictive tools are needed.

Plante et al. (2015) proposed a NMD prediction method using a modified leaching procedure on fresh and altered waste rock samples from the Lac Tio Mine that involved rinsing the material with an ethylenediaminetetraacetic acid (EDTA) solution, which has a strong complexing ability, in weathering cells. EDTA ([CH₂N(CH₂CO₂H)₂]₂) is a strong cation chelator that has been used for soil remediation since the late 1980s (Evangelista and Zownir 1988; Gluhar et al. 2020; Pociecha and Lestan 2012). Once metals are released in solution by their host minerals, they are rapidly bound by EDTA, as shown in Eq. 1, and this prevents them from precipitating or sorbing to surfaces, suppressing a leaching delay (Plante et al. 2015).

1 $$\:{H}_{2}EDT{A}^{2-}+M{e}^{2+}\to\:{H}_{2}EDTAMe$$

While H_2_EDTA^2−^ and HEDTA^3−^ are the most prevalent species of EDTA at neutral pH (which might protonate or deprotonate depending on the pH; pKa values are 0, 1.5, 2, 2.7, 6.2, 10.4)and Me^2+^ is any bivalent cation (stability constants of different metal-EDTA complexes are listed in Supplementary Table S-1). Therefore, with EDTA, it is possible to assess the “true” amplitude (worst case scenario) and rate of metal leaching. Lévesque Michaud et al. (2017) describes a study on other materials comparing the use of chelating agents such as EDTA with that of citrate (C_6_H_5_O_7_) for predicting metal leaching; citrate was used because it is biodegradable and could be applied in the field. Both studies used an excess of EDTA, which altered the mineral surfaces of more refractory phases that were not altered in control tests using deionized water. However, citrate is to be avoided in cases where iron leaching is expected, because iron citrate (solubility in water of ≈ 5 g/L, or 20 mM) easily precipitates at the citrate concentrations needed for chelating all cations in typical mine waters. Therefore, an EDTA-modified kinetic test approach that enables metal leaching while minimizing extensive dissolution of phases refractory in water is needed.

The objective of this study was to provide a general method for predicting NMD formation by building on the results of previous studies and refining the modified kinetic leaching procedure proposed by Plante et al. (2015) using Lac Tio waste rock as a positive control. The method combines modified kinetic tests with EDTA leaching and estimation of the sorption potential for Ni. Waste rock from the Lac Tio Mine site were used because it had been classified as Ni-rich (main contaminant) NMD generators.

## Materials and Methods

The Lac Tio Mine is located 43 km north of Havre-Saint-Pierre in the Côte-Nord region of Québec, Canada. The mine has been operating since the mid-1950s and exploits one of the largest known massive hematite-ilmenite deposits. There is no milling at the mine site and ore is transported by rail and barge to the Sorel-Tracy metallurgical complex in Québec, Canada. Considerable tonnage of waste rock is stockpiled at the mine site.

Some of the waste rock is known to produce Ni-NMD (Demers et al. 2013; Plante et al. 2011a, b; Poaty et al. 2021). Field observations have also shown that Ni-NMD takes years to decades to develop after exposure to atmospheric conditions (Benzaazoua et al. 2013), depending on the waste rock composition and storage conditions. The in-situ water quality is characterized by a pH ≈ 7 and Ni concentrations up to 4.5 mg/L (e.g. Poaty et al. 2021, 2022). As the Lac Tio waste rock has been extensively studied (e.g. Benzaazoua et al. 2013; Demers et al. 2011, 2013; Plante et al. 2010) and the Lac Tio Mine is still operating and generating fresh waste rock, it is an ideal source material to evaluate the method proposed in this paper as a positive control.

### Chemical, Mineralogical, and Physical Properties

Waste rock was deposited in the piles less than one year before sampling and had an average ilmenite content of 45%, which is representative of the waste rock piles’ average. The material was homogenized and sieved to < 2 cm, which is the recommended size for the column used in this study (14 cm internal diameter). A fraction of the sieved homogenized rock was sieved to < 2.5 mm and homogenized again for use in the sorption experiments. As the rock was layered for the column experiments, samples were taken after each third of the column was filled. These samples were dried separately and combined into a composite sample for each column. After homogenization, the samples were split in two, with one part stored and the other part crushed and pulverized with a Fritsch Planetary Ball Mill pulverisette 3, rehomogenized, and then split into different bags for chemical and mineralogical analysis.

The metal content was analyzed by ICP-MS after a four-acid digestion (HNO_3_, HClO_4_, HF, HCl). Total carbon and sulfur were determined using an ELTRA CS-2000 induction furnace. The samples were subjected to ABA analysis using the sulfur content to calculate the acid production potential (AP), while the neutralization potential (NP) was determined using a modified Sobek procedure NP (Lawrence and Wang 1997). The specific gravity (Gs) was determined using a Micromeritics helium pycnometer according to ASTM standard D4892. The automated SEM-EDS analysis was performed by IOS-Services Géoscientifiques (Chicoutimi, Québec, Canada) to quantify the mineral phases using a Zeiss Sigma 300 VP field emission scanning electron microscope (FE-SEM) equipped with an Ultim-Max 170 mm^2^ electron dispersive spectrometer (EDS-SD) from Oxford Instruments. Results were analyzed with the IOS-Services Géoscientifiques ARTmin in-house built program.

### Kinetic Column Leaching

The material was subjected to column leaching experiments. The column consisted of a 14-cm diameter, 90-cm high hard plastic (HDPE) tube. The bottom of the column contained a gridded base to allow water to flow gravitationally to the outlet. A double layer of geotextile was installed over the base to prevent loss of fines during flushing. Waste rock samples were placed in the column by homogeneous stacking to avoid preferential flow paths and ensure homogeneous properties.

Two columns were constructed: a control column flushed using deionized water and a second column flushed using an EDTA leaching solution. The material in each column reached a height of 70 cm with a wet mass of 19.4 kg and 19 kg, respectively, and an initial porosity of 0.5. The porosity of the columns after the experiment was 0.33, and the final height was 52 cm, due to material displacement and compaction over time. Both columns were leached and dried at room temperature (20 °C). The control column was rinsed with 1.7 L of deionized water (initial pH 6) twice a month for 197 days (6.5 months), while the EDTA column was rinsed with 1.7 L of adaptive concentrations of EDTA solution (initial pH 7.5) twice a month for 183 days (6 months). Therefore, leachate samples were collected every 14 days. The volume of leaching solution was based on previous studies that replicated the volume of average monthly rainfall representative of Havre-Saint-Pierre (Pépin 2009; Poaty et al. 2021). The initial EDTA concentration used in this study was 0.018 M, which is twice the total cation concentration in the initial leachate sample (t = 0 days) of the control column. The concentration of EDTA was doubled to completely chelate the cations expected to leach (control solution) and the excess EDTA was intended to chelate the ions not leached from the control column experiment due to potential sorption and precipitation reactions. Doubling the concentration was based on the fact that a previous experiment used 10 times more EDTA than the expected initial control cation concentration (Lévesque Michaud et al. 2017; Plante et al. 2015), but it affected the surfaces of more refractory minerals such as ilmenite. Therefore, the choice of reducing the concentration to twice the cation concentration was made to minimize the effect of EDTA on the weathering of the refractory minerals. EDTA can induce reductive dissolution of surfaces depending on the manner in which it binds to the surface (Nowack and Sigg 1996). The concentration was kept constant for the leachate samples from t = 0 days to t = 42 days to account for the first flush effect, attributed to the dissolution of efflorescent salts present at the beginning of the test, as well as reaction and depletion of the finest fraction of minerals (Maest and Nordstrom 2017; Nordstrom 2011). Because EDTA was expected to dissolve iron oxides and different precipitates than salts, the fourth leachate sample (t = 46 days) was chosen arbitrarily as the best time for concentration re-equilibration. The EDTA concentration was then modified for each subsequent leachate sample based on twice the total cation concentration in the previous leachate. For example, the EDTA concentration for the leachate sample at t = 126 days was based on the cation concentration in EDTA previous leachate sample at t = 112 days. Speciation of EDTA was also checked using Visual MINTEQ at each leachate samples to verify the excess or deficiency of EDTA and adjust accordingly in the following leachate samples.

The leaching solutions were in contact with the material for at least 4 h before being drained from the solution. Due to the grain size distribution, only the residual leaching solution was present between leachate samples, and no external control on the oxygen saturation (or water content) was applied.

Leachates were collected and analyzed for pH using a Thermo Scientific Orion Green pH combination electrode with a VWR^®^ SympHony B30PCI meter and for conductivity using a VWR^®^ Traceable^®^ Expanded Range conductivity meter. Leachates were filtered through a 0.45-µm filter. The control column samples were acidified with 2% HNO_3_ for ICP analysis, while the EDTA column samples were not acidified, because the EDTA acts as a preservative in water, and adding acid would cause a precipitate to form in the sample. The samples were analyzed on an Agilent 5800 ICP-OES for 30 elements: aluminium (Al), silver (Ag), arsenic (As), barium (Ba), beryllium (Be), bismuth (Bi), boron (B), cadmium (Cd), calcium (Ca), chromium (Cr), cobalt (Co), copper (Cu), iron (Fe), mercury (Hg), potassium (K), lithium (Li), magnesium (Mg), manganese (Mn), molybdenum (Mo), sodium (Na), nickel (Ni), lead (Pb), selenium (Se), silicium (Si), strontium (Sr), sulfur (S), tellurium (Te), titanium (Ti), uranium (U), and zinc (Zn). Values below detection limit (DL) are displayed in graphs as half of the DL.

### Batch Sorption Experiments (Kinetic Test and Isotherm)

The material used for the sorption experiment was the 2.5-mm fraction of the starting material. The solution used for the kinetic experiment was 100-mg/L Ni solution buffered to pH 6.15 with 0.1 M 2-(N-morpholino) ethanesulfonic acid hemisodium salt (MES), which also served as an ionic strength stabilizer. The initial material and the post-test dismantling material from the column leaching experiments were subjected to the batch sorption experiments by using 30 mL of the Ni-MES solution in combination with 7.5 g of material to achieve a liquid/solid ratio (L/S ratio) of 4 mL/g, which is more relevant and representative for sorption experiments (Wang et al. 2009). To achieve higher saturation of sorption sites and confirm the data obtained at a L/S ratio of 3, another test was conducted using a L/S ratio of 10 mL/g, with 3 g of material in 30 mL of the Ni-MES solution.

A kinetic sorption experiment was necessary to determine the optimum time duration for the sorption experiments. To do so, five duplicate 250-mL Erlenmeyer flasks were used, and sacrificial samples were collected at 1 h, 3 h, 6 h, 24 h, and 48 h. Samples were stirred on a rotary shaker at 200 rpm. Batch experiments for the sorption isotherm were conducted for 6 h, based on the stabilization time obtained through the kinetic experiments, and concentrations of 1, 10, 25, 50, and 250 mg/L Ni were chosen. Filtered and acidified liquid aliquots were analyzed by ICP-AES to determine Ni concentrations in solution. The following calculation (Eq. 2) was used to obtain the concentration of Ni sorbed by the material:

2 $$\:({C}_{i}-{C}_{e})\cdot\:\frac{{V}_{liquid}\:}{{M}_{solid}}={q}_{e}$$

The Langmuir isotherm can be used to plot q_e_, the amount of metal adsorbed at equilibrium in mg/kg; C_i_ is the initial concentration in mg/L, C_e_ is the equilibrium concentration in mg/L, V_Liquid_ is the volume of liquid in L, and M_solid_ is the mass of solid in g. The Langmuir isotherm equation (Langmuir 1918) is:

3 $$\:{q}_{e}=\frac{{q}_{max}{K}_{L}{C}_{e}}{1+{K}_{L}{C}_{e}}$$

where q_max_ is the maximal sorption capacity in mg/kg and K_L_ is the Langmuir constant in L/mg. The equation can be linearized in four different ways (Kumar and Sivanesan 2005; Narayanan et al. 2017; Parimal et al. 2010); see supplementary Table S-2. Among these linear forms, Langmuir 1 and 4 are considered to be best for parameter estimation of the non-linear form of the Langmuir isotherm (Guo and Wang 2019). Due to the widespread use of the Langmuir 1 linearized form, emphasis was placed on this type of linearization, as well as on a non-linear regression using the least-squares method.

The q_max_ parameter is important in this method since it allows determination of the maximum sorption capacity, which can then be compared to the total metal concentration in the material, in this case Ni. The Langmuir isotherm model is preferred to the Freundlich isotherm model because it enables the calculation of a maximum sorption capacity, whereas the Freundlich isotherm model does not, even though it may fit the data better.

### Results and Interpretation

Supplementary Table S-3 shows the initial composition of the materials prior to the column experiments. The difference between both samples can be explained by a potential heterogeneity of the waste rock samples. The average nickel concentration in the control and EDTA materials for the < 2 cm waste rocks was 430 ± 160 mg/kg (*n* = 2). These concentrations are within those obtained on the same waste rock samples in previous studies (e.g. Benzaazoua et al. 2013; Demers et al. 2011, 2013; Plante, 2011a, b). Another element with high concentrations was manganese, ranging from 650 to 800 mg/kg. The carbon and sulfur concentrations suggest that the waste rock samples have an uncertain potential for acid generation according to the NP/AP ratio and net neutralization potential (NNP) (Sobek et al. 1978).

Mineralogy.

The mineralogical modal composition determined by automated SEM-EDS analyses of the Lac Tio waste rock is shown in supplementary Table S-4. Ilmenite (including hematite exsolutions), plagioclase, enstatite, and magnetite comprise up to 95.6% of the waste rock, with the remainder being traces of apatite, pyrite, and other minerals. The main nickel-bearing mineral responsible for the NMD at Lac Tio is Ni- and Co-rich pyrite (mean of ≈ 2.1% Ni and 0.92% Co), similar to what was observed by Toubri et al. (2022) on similar materials.

### Kinetic Testing

**Fig. 1:**
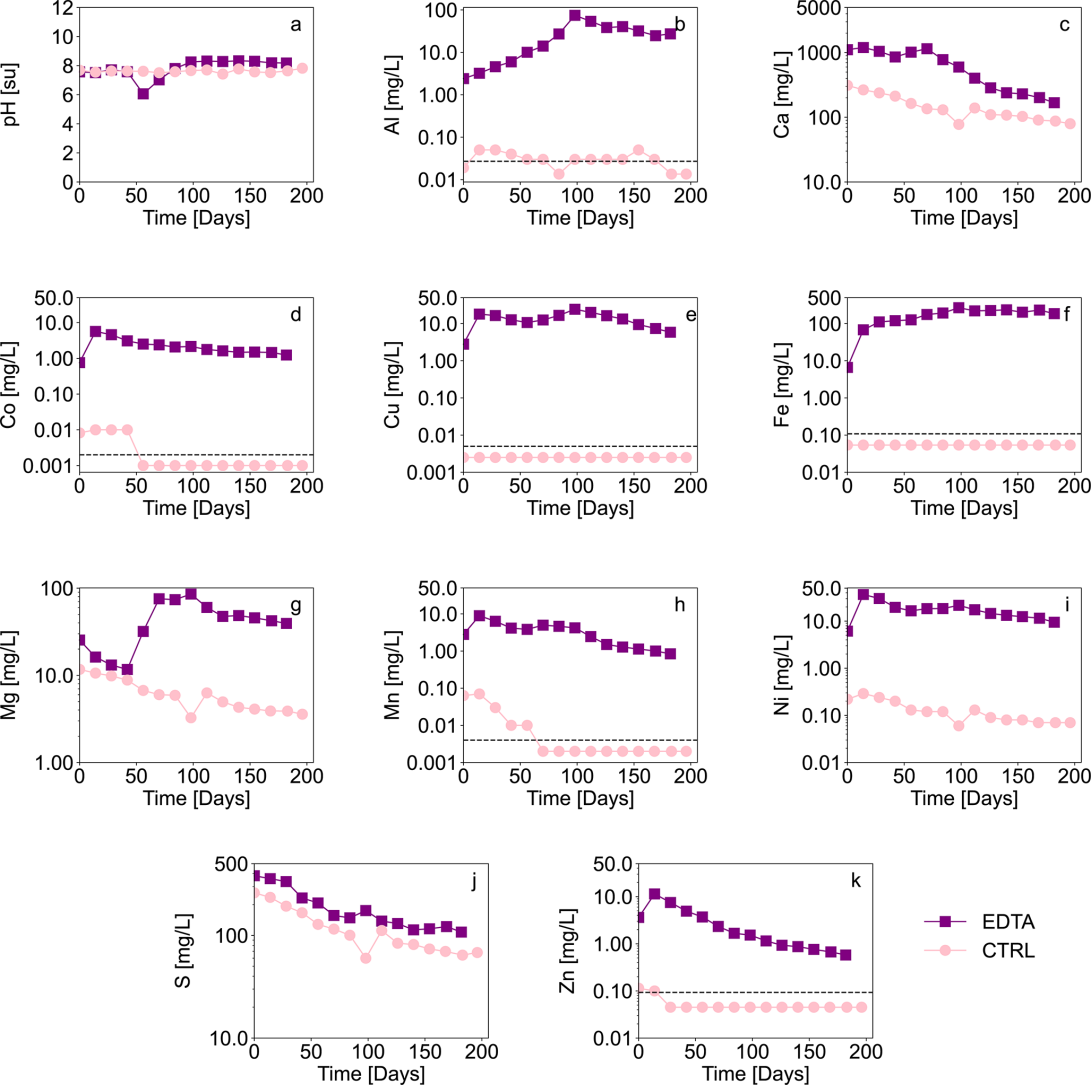
Evolution of (**a**) pH, and dissolved metal concentrations in the control (CTRL, pink dots), and EDTA experiments (purple squares): (**b**) Al, (**c**) Ca, (**d**) Co, (**e**) Cu, (**f**) Fe, (**g**) Mg, (**h**) Mn, (**i**) Ni, (**j**) S, (**k**) Zn Note the logarithmic scale for all graphs except a and b. The horizontal dashed line represents the detection limit

Figure 1 shows the evolution of pH and metal concentrations in the leachates during the column experiments. In the kinetic experiments, the pH remained neutral throughout the duration of both the control (7.4–7.8) and EDTA leaching columns (6.06–8.30) (Fig. 1a). The pH dropped in the EDTA column when the EDTA concentrations were raised after the fourth leachate sample (at t = 56). The surplus EDTA might have induced mineral dissolution that released H^+^ ions during the process or induced geochemical reactions that released H^+^; the specific cause was not investigated further and the pH returned to values > 7 in the subsequent leachate samples.

Metal concentrations in the EDTA experiment were always greater than those in the control experiment and generally decreased over time after reaching peak values. Aluminum (Al) concentrations in the control remained near the detection limit (< DL, 0.05 mg/L; Fig. 1b). However, in the EDTA experiment, the Al concentrations ranged from 2.4 to 74 mg/L (Fig. 1b). In the control experiment, the calcium (Ca; Fig. 1c) concentration was 312 mg/L at the beginning of the test and stabilized between 80 and 110 mg/L. In the EDTA column, the Ca concentration ranged from 1000 to 1200 mg/L in the leachates samples (t = 0 to t = 70 days) and gradually decreased to 168 mg/L at the end of the experiment. These results illustrate that Ca leaching is increased by the presence of EDTA, as Ca is leached from relatively reactive minerals like carbonates and Ca feldspars; these results are consistent with previous studies (Lévesque Michaud et al. (2017; Plante et al. 2015).

In the control column, Co (Fig. 1d) concentrations were slightly above the detection limit during the first rinses (0.01 mg/L) and then decreased to 0.002 mg/L. In the EDTA experiment, cobalt was detected at 5.7 mg/L and gradually decreased to 1.2 mg/L. Copper concentrations (Fig. 1e) showed a similar trend as Co, with concentrations below the detection limit (< 0.005 mg/L) in the control and increasing concentrations ranging from 18 to 10 mg/L in the first five leachate samples (from t = 0 to t = 56 days). The subsequent increase in Cu concentration to 24 mg/L suggests that either the EDTA concentration was not high enough to chelate all the ions and complexes with those for which it has a higher affinity, or that the increased EDTA concentration leached more Cu-containing minerals. Thermodynamic equilibrium calculations (not shown) suggested that Cu precipitated as secondary oxyhydroxides in the control column. Therefore, since Cu oxyhydroxides are sparingly soluble at near-neutral pH (Baltpurvins et al. 1996; Eriksson and Destouni 1997; Kim et al. 2008), the Lac Tio waste rocks will likely not leach significant Cu, as long as the drainage pH remains circumneutral.

In the control experiment, Fe concentrations (Fig. 1f) remained below the detection limit of 0.1 mg/L. Fe oxyhydroxides are likely to precipitate at neutral pH in the control column (e.g. Bigham and Nordstrom 2000; Cravotta 2008). However, in the EDTA experiment, Fe concentrations increased from 6.6 to 267 mg/L and remained around 200 mg/L after the leachate sample at t = 96 days. Plante et al. (2015) demonstrated that EDTA can leach Fe from iron oxides, such as ilmenite. Mineralogical observations were made at the end of the experiment, and no “trellis” pattern, typical of ilmenite weathering (Frost et al. 1983; Nair et al. 2009; Plante et al. 2015), was observed in ilmenite grains on the EDTA-leached material, suggesting no excessive leaching. Therefore, it is likely that the Fe released was mostly from sulfide oxidation and the dissolution of secondary Fe-oxyhydroxides and amorphous phases at concentrations too low to be detected at the start of the experiment.

The concentrations of Mg (Fig. 1g) in the control decreased from 11 mg/L to 3.6 mg/L during the experiment. However, Mg exhibited the greatest change in the EDTA experiment. Initially, Mg levels ranged from 13 to 32 mg/L when the EDTA concentration was the lowest. When the EDTA concentration was increased, Mg concentration increased by 2 to 3 times, peaking at 85 mg/L, and then decreased to 39 mg/L. The sudden rise in concentration can be attributed to the fact that Mg has the lowest stability constant for its complexation with EDTA within the elements analyzed (i.e. the lowest affinity), and therefore EDTA was chelating other metals before Mg until there was sufficient EDTA to chelate the Mg (Lévesque Michaud et al. 2017; Smith et al. 2004) (See supplementary Table S-1 for affinity constants of EDTA and cations), which happened much more when EDTA concentration was increased. Manganese concentrations (Fig. 1h) in the control leachates were detectable until the leachate sample at t = 56 days (0.07 − 0.01 mg/L), after which they fell below the detection limit (0.004 mg/L). In the EDTA experiment, concentrations peaked at 8.9 mg/L and decreased to 0.84 mg/L over time (Fig. 1j).

In the control column, Ni concentrations (Fig. 1i) began to leach at 0.22 mg/L and decreased to 0.070 mg/L over time. In the EDTA experiments, Ni concentrations initially reached up to 37 mg/L and decreased to 9.5 mg/L after 182 days. Sulfur concentrations (Fig. 1j) were comparable in the control and EDTA experiments, with somewhat greater levels in the EDTA phase (381 mg/L–107 mg/L S in EDTA vs. 259 mg/L–64 mg/L S in the control). Zinc concentrations (Fig. 1k) exhibited a similar pattern to cobalt, mostly below the detection limit in the control and reaching concentrations between 11 mg/L and 0.57 mg/L in the EDTA experiment.

**Fig. 2:**
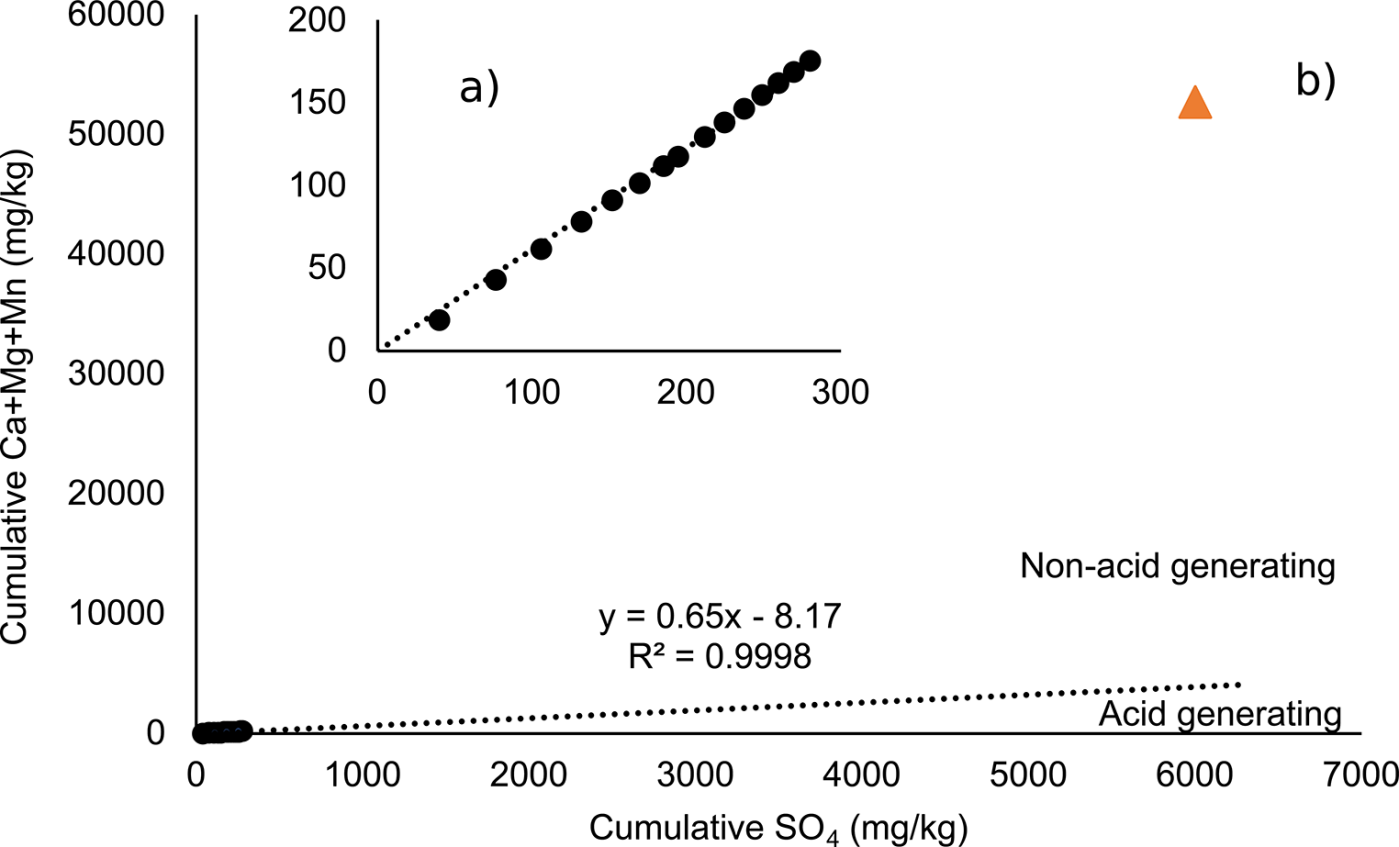
(**a**) Experimental data of cumulative subsequent sulfate and cumulative Ca + Mg + Mn loadings [mg] normalized to the mass of rock [kg]in the control kinetic experiment (inset) and (**b**) projection of the Lac Tio control waste rock sample (mean values of the initial two samples) on the extrapolation of the neutralization curves

Figure 2a and b depict an inset (Fig. 2a) and an extrapolation of the oxidation-neutralization curve (Fig. 2b) using cumulative loadings of subsequent leachate samples from the control kinetic tests normalized by waste rock mass from the control column experiment. The oxidation-neutralization curve is representative of sulfide oxidation to sulfate (and subsequent acid generation) and acid consumption by neutralizing minerals (which typically consume the acidity and release calcium, magnesium, and manganese as a result (Benzaazoua et al. 2004). The projection of the Ca + Mg + Mn and the S initial solid content on the graph allows for the prediction of whether the rock is prone to acid generation. If the initial solid content of Ca + Mg + Mn vs. SO_4_ is located above the projected curve (Fig. 2b), the material will have enough neutralizing material to compensate for the acidity generated by sulfide oxidation. However, if the initial solid content of Ca + Mg + Mn vs. SO_4_ is located below the projected curve, the material will end up using all of the neutralizing minerals before complete depletion of sulfide minerals and AMD occurs. The projection of the average Lac Tio waste rock sample results suggests that the sample is non-acid-generating as the Ca + Mg + Mn vs. SO_4_ is located above the extrapolation of the oxidation-neutralization curve.

### EDTA Concentrations During Kinetic Testing

The concentration of EDTA was kept as low as possible to avoid problems of over-alteration of oxide phases, as observed in Plante et al. (2015). Therefore, the EDTA concentration was adjusted during the kinetic experiment to remain as close as possible to twice the concentration of cations in the EDTA leachate samples (as opposed to 10 times the concentration in previous studies). This was done on the assumption that one mole of EDTA chelates one mole of cations, and that there should be a slight excess to chelate everything that can be chelated, but not so much as to attack mineral surfaces.

**Fig. 3:**
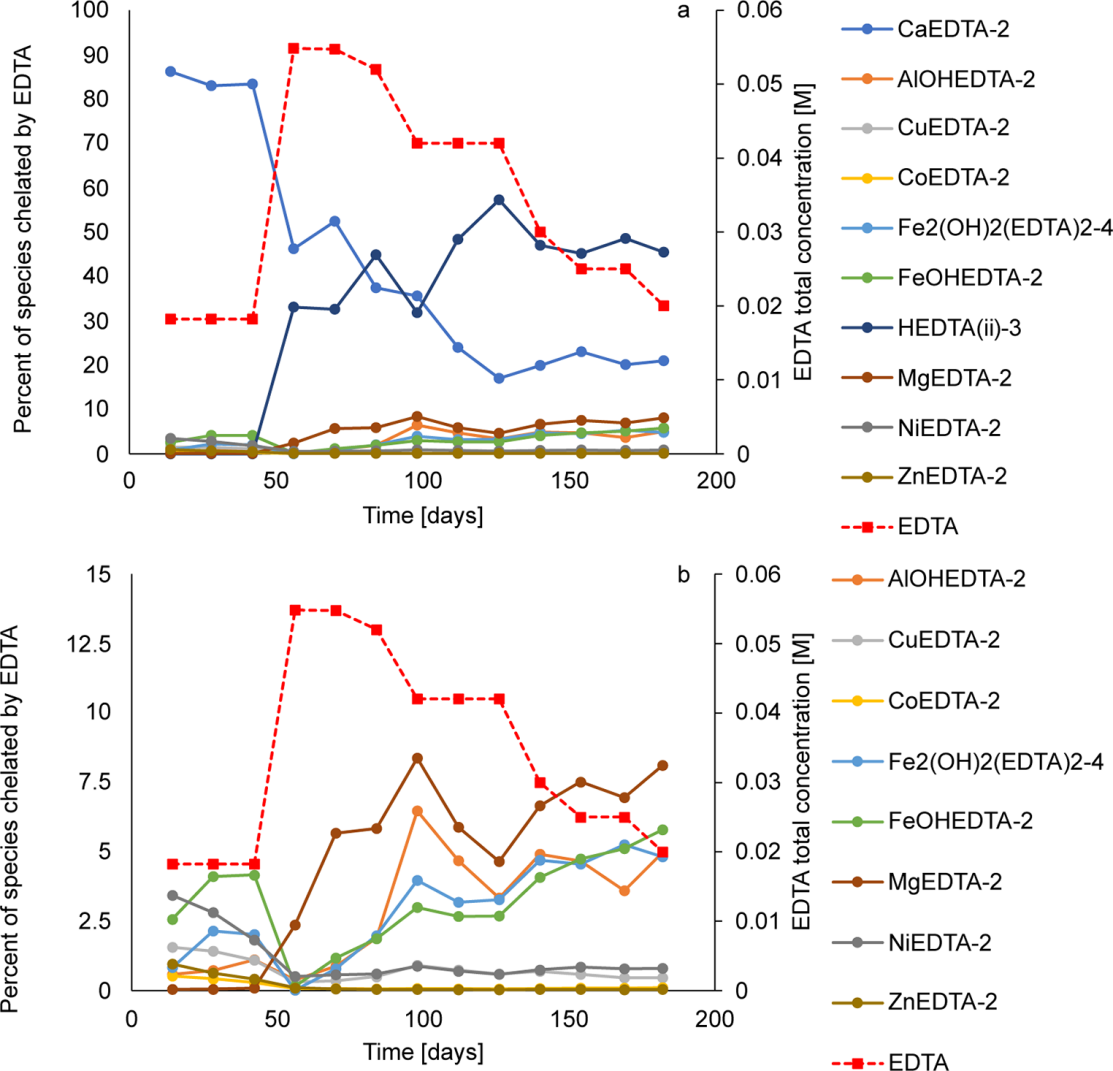
Evolution of EDTA speciation during column testing with EDTA (total EDTA concentrations shown in red squares, see right y axis): (**a**) Calcium and unchelated H-EDTA form (dominant form of EDTA at neutral pH) with other species, (**b**) species (other than Ca) that occupy less than 10% of the EDTA

Figure 3a and b depict the EDTA complexation during the ETDA kinetic experiment. The initial EDTA leachate sample (t = 0 days) is not shown because Visual Minteq was not able to calculate the equilibrium constants in the initial EDTA sample. The modified kinetic experiments were started with a concentration of 0.018 M EDTA, which is twice the cation concentration measured in the initial leachate at t = 0 days of the conventional control test, based on the hypothesis that twice the cation concentration in the control could potentially be leached with EDTA chelation. This EDTA concentration was maintained for leachate samples up to (and including) t = 42 days. After the leachate sample at t = 42 days, the EDTA concentration was re-evaluated because the cation concentration of the fourth flush was less affected by the “first flush effect” that generally occurs within the first leachate samples (e.g. Maest and Nordstrom 2017; Nordstrom 2009). This time, EDTA concentration was calculated as twice the cation concentration in the EDTA leachate sample at t = 42 days. Therefore, for the leachate sample at t = 56 days, the total EDTA concentration was increased to 0.054 M because the concentration of cations was greater than what the EDTA could chelate in the leachate sample at t = 42 days. This can be seen in Fig. 3a, where free EDTA species (and HEDTA^− 3^) do not represent a significant fraction of the EDTA species in leachates samples from t = 14 days to t = 42 days. However, after t = 42 days, the proportion of free EDTA (HEDTA^− 3^) increased (Fig. 3a), the proportion of CaEDTA^−^ decreased (Fig. 3a), and the proportions of Fe-, Mg- and Al-EDTA complexes increased (Fig. 3b). This confirms that adjusting the EDTA concentration promoted the leaching of Fe, Mg, and Al, which were not fully chelated before the adjustment due to a lack of EDTA (Fig. 3b). After t = 70 days, the total EDTA concentration was decreased gradually to maintain the free EDTA (HEDTA^3−^) between 40% and 60% of the overall EDTA species (Fig. 3a). This range was chosen arbitrarily, with the aim of having a slight excess of EDTA relative to the overall cation concentrations in the EDTA leachate samples.

**Fig. 4:**
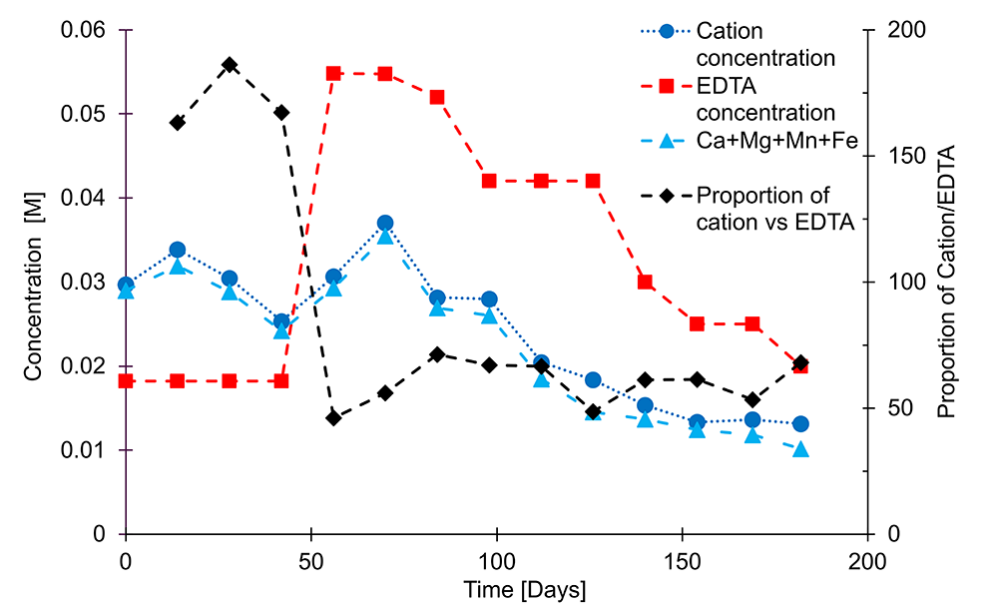
On the left y axis: total EDTA molar concentrations (red squares), total molar concentration of cations (without Na, which is present in the EDTA salt) (dark blue circles) and Ca + Mg + Mn + Fe molar concentrations (light blue triangles). On the right y axis, proportion of total cations chelated with EDTA (black diamonds)

Figure 4 displays that there was not enough EDTA to chelate the leached cations (ratio of cation to EDTA > 100%). It decreased when the EDTA concentration was increased to accommodate the excess cation. It also shows that VMinteq is not necessary to calculate the EDTA concentration needed to chelate the cations. In fact, the EDTA concentration could have been adjusted from the total cation concentration (blue circles). In addition, a further simplification could be made by adding the major cations chelated by EDTA, namely Ca, Mg, Mn and Fe.

### Leaching Rates

The EDTA increased the leaching rate of all ions except sulfur by several orders of magnitude compared to the control. Indeed, the leaching rates of all cations increased because EDTA prevented their immobilization by sorption and secondary precipitation, resulting in their leaching (Table 1). However, the sulfur leaching rate increased by a factor of only 1.5 over the course of the experiment. This result was expected because the EDTA prevents the accumulation of secondary oxyhydroxides on the sulfide surfaces, optimizing the available surfaces for oxidation (Plante et al. 2015; Rumball and Richmond 1996) and also prevents the precipitation of sulfate minerals due to the chelation of cations by the EDTA. In the post-dismantlement samples, it was observed that sulfides did not accumulate Fe-oxyhydroxide coatings over time (see next section) due to the chelating power of EDTA with Fe, resulting in an oxidation rate slightly higher in the EDTA test than in the control.

**Table 1:** Leaching rates in the control and the EDTA experiment

Element	Leaching Rate CTRL [mol kg^− 1^day^− 1^]	Leaching Rate EDTA [mol kg^− 1^day^− 1^]	EDTA/control [ - ]
Ca	2.1E-5	3.8E-5	4.57
Mg	1.5E-6	1.1E-5	7.33
Fe	5.7E-9	1.9E-5	3333
S	2.2E-5	3.5E-5	1.59
Cu	2.3E-10	1.3E-6	5652
Ni	1.3E-8	1.8E-6	138
Mn	1.5E-9	3.7E-6	2467
Al	7.1E-9	5.9E-6	831
Zn	4.8E-9	2.7E-7	56.3
Co	7.2E-10	5.0E-7	694

Next to the release rates of S, the release rates of Ca, Mg, and Zn were the least affected by the EDTA leaching procedure, whereas Fe, Cu, and Mn were highly influenced by the EDTA leaching procedure. This suggests that the EDTA prevented the immobilization of those elements (by precipitation and sorption).

### Impact of EDTA on Mineral Surfaces

EDTA is known to enhance weathering of mineral phases when used in excess (Lévesque Michaud et al. 2017; Plante et al. 2015). EDTA could also weather surfaces of more refractory minerals that are unlikely to weather in regular leaching conditions, leading to an increase in ion release. In this study, an attempt was made to adjust the proportion of free EDTA between 40 and 60% of the total EDTA concentration to ensure sufficient chelation of additional ions while minimizing excessive weathering; microscopic observations made on the post-dismantlement materials suggest the success of this approach.

**Fig. 5:**
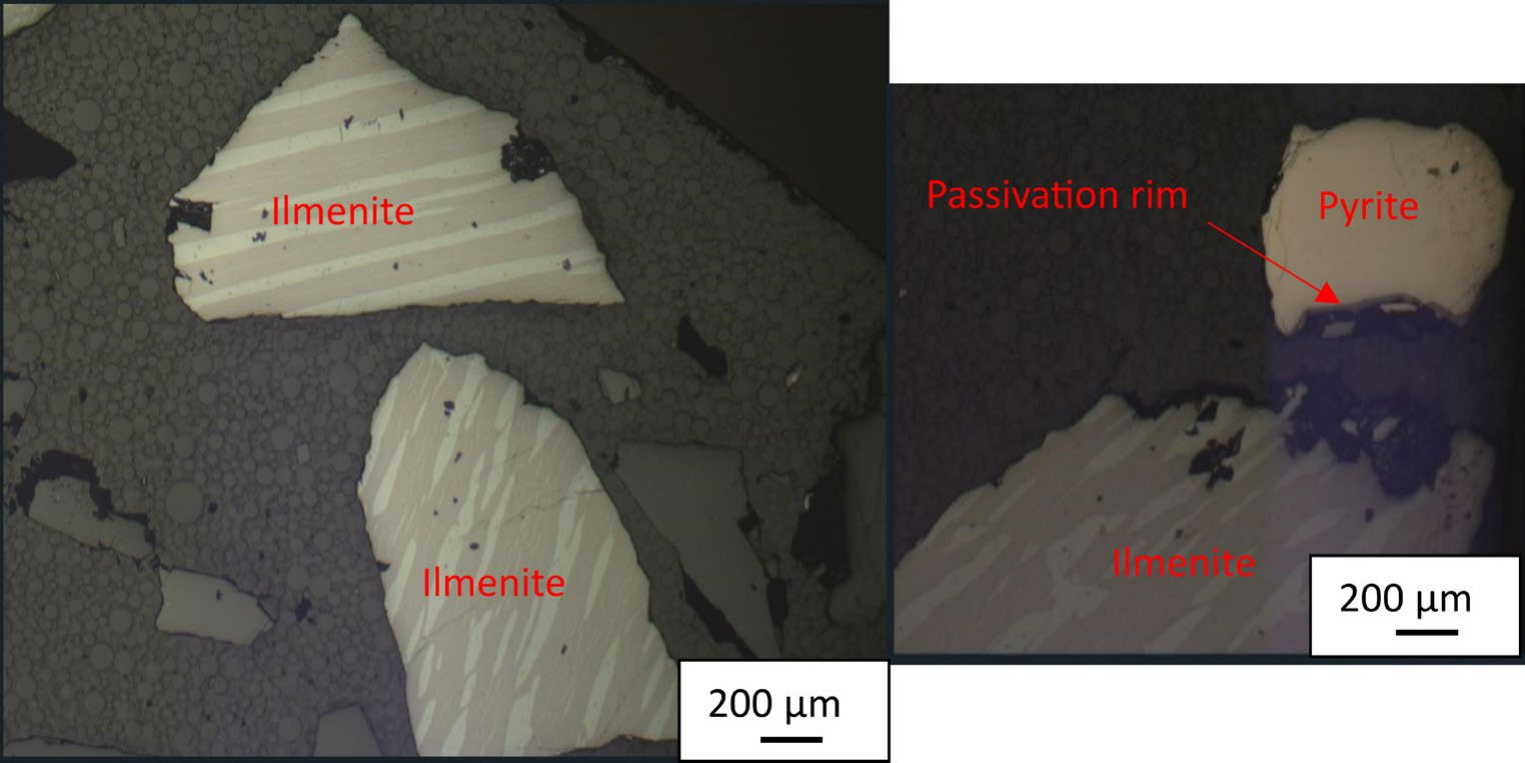
Mineralogical observations in a sample from the EDTA experiment with unaltered ilmenite (no “trellis” pattern observed) and slightly altered pyrite; scale bars are 200 µm

Observations were made of ilmenite grains due to the tendency of excess EDTA to promote ilmenite weathering, forming trellis textures on ilmenite surfaces. In this case, the ilmenite remained devoid of such textures even after six months of bi-monthly EDTA leaching. Some pyrite particles showed a slight passivation rim, typical of sulfide weathering, suggesting that the EDTA concentration was high enough to prevent the formation of a complete passivation rim, but still allowing some precipitates to form on the pyrite surface (Fig. 5). It is also possible that the slight passivation rims observed were remnants of passivation rims already present at the beginning of the column test and were partially dissolved by the EDTA.

### Cumulative Molar Loadings

**Fig. 6:**
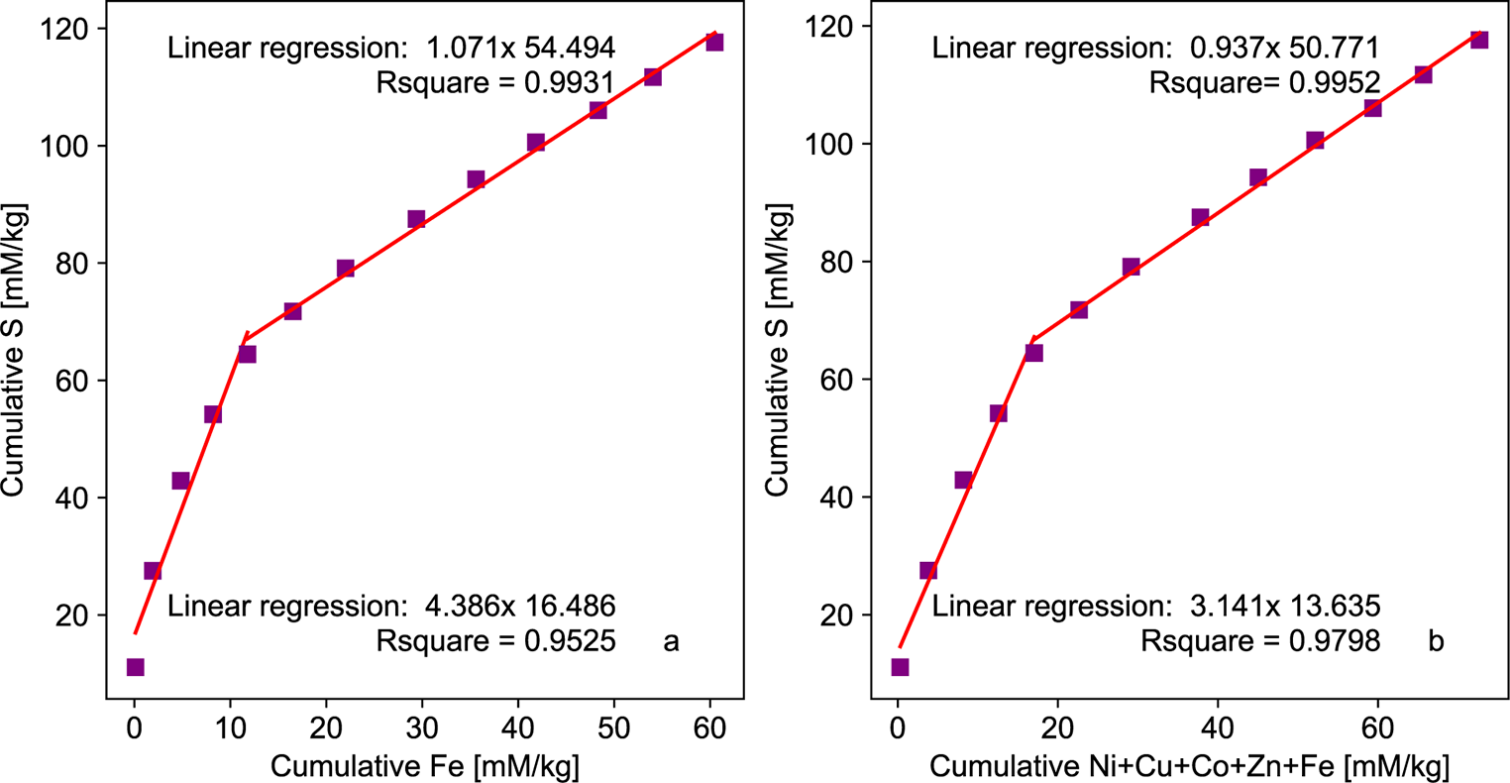
Cumulative loadings per kg of rock in the EDTA experiment, showing the relationship between (**a**) Fe and S and (**b**) Fe + Ni + Cu + Co + Zn and S

According to classical oxidation processes, pyrite (FeS_2_) is expected to react congruently, releasing one mole of Fe for every two moles of S. However, pyrite from the Lac Tio waste rock contains impurities, such as Ni, Cu, Co, and Zn, which either substitute for Fe or are contained in other sulfides in trace levels as inclusions, such as chalcopyrite (CuFeS_2_) and sphalerite (ZnS). As a result, it is expected that sulfide oxidation will release between one to two moles of metals (Fe + Ni + Cu + Co + Zn) for every mole of sulfur (depending on the sulfide). Figure 6a shows the change in the Fe: S ratio and the change in the metal: S ratio in the EDTA experiment (Fig. 6b). After the leachate sample at t = 46 days, the concentration of EDTA was increased, which resulted in an increase in the concentrations of Fe and other metal cations. This caused a shift in the S/Fe ratio from 4.4 to 1.1 (Fig. 6a), and from 3.1 to 0.9 when considering all of the metals (Fig. 6b). The first part of the graph might not be representative of a stochiometric dissolution because the EDTA concentrations are not high enough to complex all of the ions (including iron).

The S/Fe and S/metal trends during the first four flushes were above the expected ratio of two (for pyrite), indicating that the Fe was not leached as congruently as the S. Indeed, Fe might be adsorbing or precipitating because there was not enough EDTA to chelate the metals released. However, increasing the concentration of EDTA resulted in more complete chelation and leaching of the metals. In general, metals were leached in the same molar ratio as the sulfur (1 mol of sulfur released for 1 mol of metals), which was less than the ratio of two expected since pyrite is the dominant sulfide. Although no evidence of ilmenite weathering by EDTA was observed, it cannot be completely ruled out. It is possible that the Fe that leached from the ilmenite could not be chelated initially due to a lack of EDTA and was released by the subsequent addition of EDTA (and the increase in free EDTA concentration).

### Sorption Isotherms

**Fig. 7:**
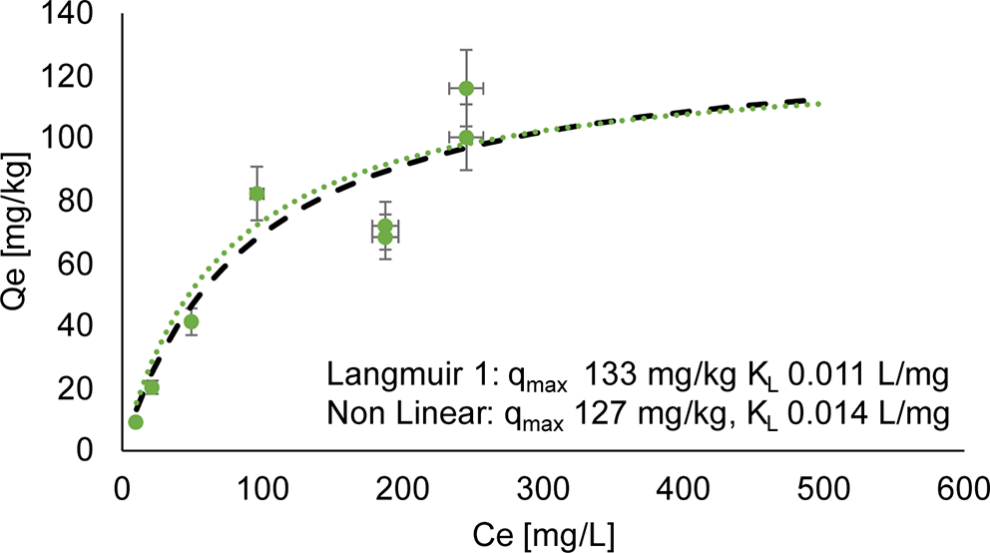
Langmuir 1 (dotted green), non-linearized (black dashes) sorption isotherms and experimental points (individual points) on initial material before column experiments, L/S 4

**Fig. 8:**
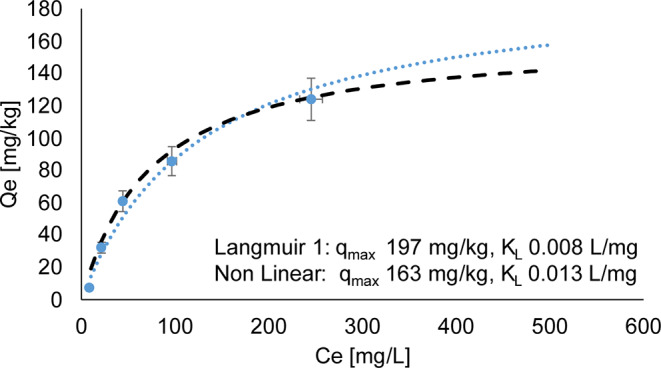
Langmuir 1 (dotted blue), non-linearized (black lines) sorption isotherms and experimental points (individual points) on initial material before column experiments, L/S 10

**Fig. 9:**
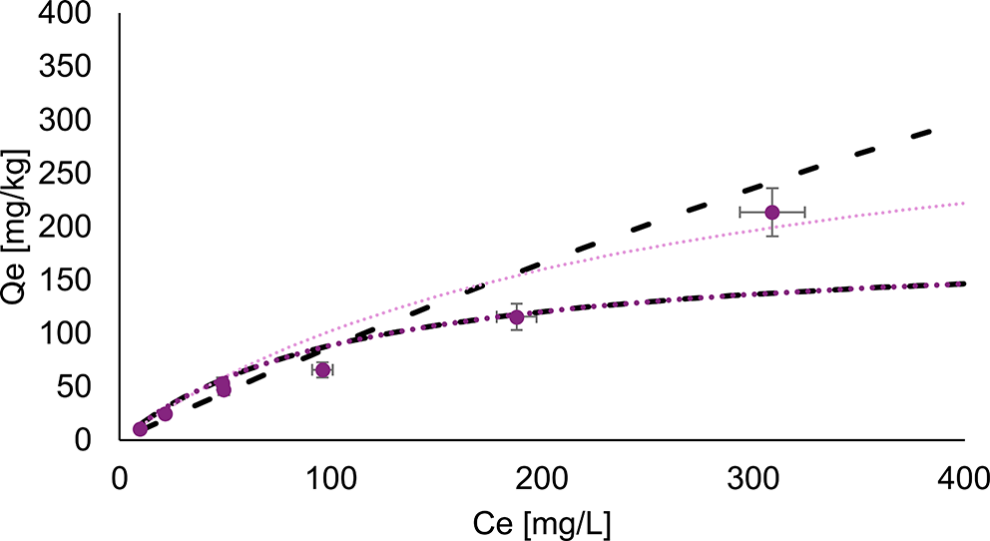
Langmuir 1 isotherm excluding the outlier at equilibrium concentration (C_e_) = 309 mg/L (dotted medium purple dotted line), q_max_ 187 mg/kg and K_L_ 0.009 L/mg, which is overlaying the non-linearized isotherm excluding the outlier(black dashed line), q_max_ 194 mg/kg and K_L_ 0.008 L/mg. Langmuir isotherm-1 including the outlier (light purple dotted lines), q_max_ 363 mg/kg and K_L_ 0.004 L/mg, and non-linearized sorption isotherms including the outlier (black dotted and dashed line), 1534 mg/kg and K_L_ 0.008 L/mg. Experimental points (individual points) on the post-dismantlement column material

Batch sorption experiments were conducted on the < 2.5-mm fraction of both the initial and final waste rock material from the control experiment. The Langmuir isotherms for the initial waste rock with a L/S ratio of 4 and 10 are displayed respectively in Figs. 7 and 8, while Fig. 9 shows the isotherms for post-dismantlement column samples at a L/S ratio of 4.

At a L/S ratio of 4, the linearized isotherm suggests a maximum sorption capacity of 133 mg/kg, while the non-linear Langmuir isotherm showed a capacity of 127 mg/kg (Fig. 7). A slightly higher maximum sorption capacity was found for the same waste rock sample with a L/S ratio of 10, namely 197 mg/kg for the linearized isotherm and 163 mg/kg for the non-linearized isotherm (Fig. 8). The values from the two L/S ratios are similar in magnitude.

The isotherms on the post-dismantlement material (Fig. 9) shows one data point that could be considered an outlier at C_e_ = 309 mg/L. One potential explanation for the outlier might be that, at higher concentrations observed in the solution, the Langmuir model might not be ideal. Indeed, the Langmuir model considers homogeneous sorption sites organized in a single layer. However, at high enough concentrations, multiple layers of sorption sites can develop. Although the Freundlich model considers multiple layers and might be better suited at these higher concentrations, it does not provide the q_max_ value needed for the present study, and it was therefore decided to use the Langmuir model despite its limitation. A linearized isotherm was performed on the data, both including and excluding this potential outlier. The resulting q_max_ values were 187 mg/kg when excluding the point and 363 mg/kg when including it. When applying the non-linear Langmuir isotherm, the q_max_ value was 194 mg/kg excluding the potential outlier and 1519 mg/kg including it. It is possible that the sorption capacity had increased over time due to potential precipitation of oxides upon weathering, which tends to increase the sorption capacity, but an increase as important as what is suggested by the non-linearized isotherm including the potential outlier seems unlikely, given that it is the only method that predicts such a high value. To remain conservative in the NMD risk assessment, this higher value will not be considered.

The total amount of Ni leached from the EDTA column, normalized to the mass of material in the column, was calculated to be 42 mg/kg. Thus, should the maximum sorption capacity remain unchanged despite weathering, the sorption capacity of the post-dismantlement material from the control column should decrease by ≈ 42 mg/kg compared to its initial capacity. However, the sorption capacity was not markedly changed by weathering. These results demonstrate that the sorption capacity evolution over time needs to be investigated further. The potential saturation of sites and the generation of new sorption sites are dynamic processes and might cancel each other. Although predicting the changes in sorption capacity over time seems challenging, assuming that it remains as determined on the initial material is the best approach that should be used when assessing the risk of NMD generation due to the lack of knowledge on the evolution of sorption sites in time.

Comparatively, Plante et al. (2010) studied the sorption of Ni on Lac Tio waste rocks and found maximal sorption capacities of ≈ 1000 mg/kg for fresh rocks. The higher capacity than the present study can be attributed to a finer particle size (< 500 μm), a pH that was fixed at the start of the experiment (although it may have changed during the experiment), and a sorption time of 72 h, which allowed for a longer contact time with the surface.

The waste rock samples showed variability in the Ni concentrations, ranging from 270 to 590 mg/kg. The linearized isotherm interpretation suggests maximal sorption capacities between 127 and 197 mg/kg for the initial material. Therefore, the ratio between the highest Ni concentration (590 mg/kg) and the highest initial sorption capacity (197 mg/kg) is 3, which suggests that there were not sufficient sorption sites for the Ni potentially released. This ratio increases to 4.6 when considering the lowest sorption capacity measured (127 mg/kg). In addition, competition for Ni sorption sites by other cations potentially released, such as Co and Zn, decreases the ratio even more (assuming they are competing for the same sorption sites), which suggest an even greater risk of NMD generation. This shows that using the Langmuir isotherms enables the prediction of the risk of Ni leaching from fresh Lac Tio waste rocks. Thus, considering the uncertainties surrounding Ni concentrations and sorption capacity, the described methodology can be used to predict the risk of Ni leaching in Lac Tio waste rocks.

### Integration of Kinetic and Sorption Results as an Assessment of the Risk for NMD

Ni-leaching rates were estimated at 1.8E-6 mol/kg/day, equivalent to 0.105 mg/kg/day, through the EDTA column experiment. Considering that the highest estimated initial waste rock sorption capacity was 197 mg/kg, should the Ni leaching rate remain constant and sorption sites gradually saturate, it would take ≈ 5 years (1876 days) to reach saturation at the conditions of the column test. However, assuming a sorption capacity of 127 mg/kg, it would take ≈ 3.3 years (1210 days) to reach saturation at these conditions.

Although Co, Mn, and Zn displayed lower leaching rates, they may also contribute to sorption site saturation. If their leaching rates are combined to that of Ni, the total is equivalent to 0.152 mg/kg/day of metals competing for the Ni sorption sites. Based on the highest estimated sorption capacity of 197 mg/kg, it would take ≈ 3.5 years (1296 days) to saturate the sorption sites in the conditions of the column study, whereas with the lowest estimate of 127 mg/kg of sorption capacity, it would saturate in ≈ 2.3 years (836 days).

## Conclusion

This study examined a method for predicting neutral mine drainage using waste rock that produced Ni-NMD over time as a confirmation material for the method’s effectiveness. The method combined quantifying metal concentrations with assessing the mineralogy to determine the association of metals in the mineral matrix and to identify potentially leachable metals. Additionally, a modified kinetic leaching experiment using EDTA as a chelator was conducted to estimate the leaching rate of metals, especially Ni in this context, without immobilization phenomena such sorption and precipitation. Finally, sorption experiments were performed to estimate the maximum sorption capacity of Ni by the waste rock itself, which is often the primary medium for retention in the field.

Overall, the results indicate that:

- The concentrations of Ni in the waste rock samples ranged from 270 to 590 mg/kg.
- The Ni was primarily present in the pyrite, which oxidized under atmospheric conditions.
- Kinetic tests with adaptive EDTA concentrations showed that Ni was leached, indicating that EDTA prevented the immobilization of Ni within the waste rock.
- The waste rock’s initial Ni sorption capacity was found to range from 127 to 197 mg/kg.
- The comparison of the initial concentration of Ni and its sorption capacity suggested a smaller number of sorption sites compared to the Ni content in the waste rock, indicating a risk of NMD.
- The leaching rates from the column experiment can be used to assess the time to saturation of sorption sites, which was to 3.3 to 5 years in the context of the column experiment, assuming that the sorption sites were occupied by only Ni.
- Competition for Ni sorption sites by other potentially leachable metals like Co and Zn decreased over time until the sorption sites were saturated (2.3 to 3.5 years).

This information was consistent with previous studies and the method proved effective for the Lac Tio waste rock. However, further testing is required to validate the method on other materials, including those that produce metallic cations like Zn, Mn, and Co, as well as materials that generate oxyanions as contaminants at neutral pH, such as Sb, As, Cr, and Mo. Further tests are also necessary to more precisely address the link between the concentration of EDTA and the potential weathering of more refractory minerals (or increased leaching of more soluble minerals). More research is also needed to understand the source of the excess of ions such as Fe as a comparison to S if stoichiometric dissolution is considered. To enhance the accuracy of the NMD prediction method, it may be necessary to consider the role of particle size distribution and liberation, the degree of oxygen saturation and temperature, as well as a more accurate quantification of the material’s sorption capacity. The sorption capacity of the material can be influenced by competitive sorption mechanisms, pH variation over time, and temperature fluctuations. This work will help practitioners manage risks associated with the generation of neutral mine drainage.

## Electronic Supplementary Material

Below is the link to the electronic supplementary material.

Supplementary Material 1
