# Supplementary material for: Development of a Neutral Mine Drainage Prediction Method Using Modified Kinetics Tests and Assessment of Sorption Capacities
Source: Mine Water Environ. 2025 Jan 21;44(1):16–29. doi: 10.1007/s10230-025-01023-6 (PMC11933128; doi:10.1007/s10230-025-01023-6)
Supplement: Supplementary file 1 — Supplementary Material 1 [file 10230_2025_1023_MOESM1_ESM.docx]

Supplementary Tables

**Supplementary Table S-1.** Stability constants (log K) of EDTA with cations for ions at 20°C and 0.1M (Lévesque Michaud 2016; Smith et al. 2004)

| Element | Stability constant  (Log K) |
| --- | --- |
| Mg | 8.69 |
| Ca | 10.7 |
| Mn | 13.6 |
| Fe^2+^ | 14.3 |
| Fe ^3+^ | 25.7 |
| Al | 16.1 |
| Co | 16.2 |
| Cd | 16.5 |
| Zn | 16.5 |
| Pb | 18.0 |
| Ni | 18.6 |
| Cu | 18.8 |

**Supplementary Table S-2.** Linearized Langmuir isotherms from Guo and Wang (2019) and references therein

| Model | Equation |
| --- | --- |
| Langmuir 1 | $\frac{C_{e}}{Q_{e}}=\frac{1}{q_{max}}C_{e}+\frac{1}{K_{L} q_{max}}$ |
| Langmuir 2 | $\frac{1}{Q_{e}}=\frac{1}{C_{e}}\frac{1}{K_{L} q_{max}}+\frac{1}{q_{max}}$ |
| Langmuir 3 | $Q_{e}=\frac{Q_{e}}{C_{e}}\frac{1}{K_{L}}+q_{max}$ |
| Langmuir 4 | $\frac{Q_{e}}{C_{e}}=K_{L} Q_{e}+q_{max}K_{L}$ |

**Supplementary Table S-3.** Material chemical and physical characteristics

| Element | Unit | Initial CTRL | initial EDTA |
| --- | --- | --- | --- |
| Aluminum | % | 5.2 | 6.0 |
| Calcium | % | 2.7 | 3.0 |
| Cobalt | mg/kg | 91 | 83 |
| Chromium | mg/kg | 920 | 430 |
| Copper | mg/kg | 79 | 100 |
| Iron | % | 23 | 18 |
| Potassium | % | 3.0 | 3.4 |
| Magnesium | % | 2.4 | 2.3 |
| Manganese | mg/kg | 800 | 650 |
| Sodium | % | 1.5 | 1.7 |
| Nickel | mg/kg | 590 | 270 |
| Silica | % | 15.4 | 14.1 |
| Sulfur | % | 0.12 | 0.13 |
| Zinc | mg/kg | 89 | 110 |
| Carbon | % | <0.05 | <0.05 |
| Sulfur | % | 0.17 | 0.23 |
| AP | mg CaCO_3_/kg | 5.3 | 7.3 |
| NP Sobek | mg CaCO_3_/kg | 7.6 | 7.4 |
| NP/AP Sobek |  | 1.4 | 1.0 |
| NNP Sobek | mg CaCO_3_/kg | 2.3 | 0.1 |
| Gs | g/cm^3^ | 3.57 | 3.44 |
| Average D_10_ | mm | 0.2 (n=2) | |
| Average D_50_ | mm | 4 (n=2) | |
| Average D_90_ | mm | 11 (n=2) | |

**Supplementary Table S-4.** Material mineralogical characteristics

| Mineral Name | SEM %wt. |
| --- | --- |
| Hemo-ilmenite | 55.8 |
| Plagioclase | 24.0 |
| Enstatite | 7.9 |
| Magnetite | 7.3 |
| Apatite | 0.9 |
| Pyrite (Ni, Co-Traces) | 0.7 |
| Other | 3.4 |
